# The Burden of Disease in Alopecia Areata: Canadian Online Survey of Patients and Caregivers

**DOI:** 10.2196/39167

**Published:** 2022-10-06

**Authors:** Anthony Justin Gilding, Nhung Ho, Elena Pope, Cathryn Sibbald

**Affiliations:** 1 Department of Chemistry and Biology Faculty of Science Toronto Metropolitan University Toronto, ON Canada; 2 Canadian Alopecia Areata Foundation King City, ON Canada; 3 Division of Dermatology Department of Paediatrics The Hospital for Sick Children Toronto, ON Canada; 4 Department of Paediatrics University of Toronto Toronto, ON Canada

**Keywords:** alopecia areata, quality of life, burden of disease, alopecia, QoL, burden, dermatology

## Abstract

**Background:**

Alopecia areata (AA) is associated with negative impacts on the quality of life (QoL). Data on this impact are lacking for Canadian patients and their caregivers.

**Objective:**

This study aims to investigate the burden of AA on Canadian patients and their caregivers.

**Methods:**

We created 4 online surveys for patients 5-11 years old, 12-17 years old, and ≥18 years old and for caregivers of children (<18 years old) with AA. These were disseminated through the Canadian Alopecia Areata Foundation (CANAAF) website and to dermatologists across Canada.

**Results:**

In total, 115 adult patients (n=100, 87%, female), 14 pediatric patients (n=13, 92.9%, female), and 15 caregivers completed the surveys online. The majority (n=123, 95%) of patients felt uncomfortable or self-conscious about their appearance. Camouflaging hair loss with hats, scarves, and hairpieces was a common practice for 11 (78.6%) pediatric and 84 (73%) adult patients. Avoidance of social situations was reported by 8 (57.1%) pediatric and 75 (65.2%) adult patients. Constant worry about losing the achieved hair growth was a concern for 8 (57.1%) pediatric and 75 (65.2%) adult patients. On a scale of 1-5, the mean score of caregivers’ own feelings of sadness or depression about their child’s AA was 4.0 (SD 0.9) and of their feelings of guilt or helplessness was 4.2 (SD 1.2). The impact on the QoL was moderate for both children and adults. Based on the Adjustment Disorder New Module-20 (ADNM-20), 71 (61.7%) of 115 patients were at high risk of an adjustment disorder. Abnormal anxiety scores were recorded in 40 (34.8%) patients compared to abnormal depression scores in 20 (17.4%) patients.

**Conclusions:**

This study confirmed a significant burden of AA on Canadian patients’ and caregivers’ QoL.

## Introduction

### Background on Alopecia Areata

Alopecia areata (AA) is an autoimmune disease affecting the hair follicles that presents with nonscarring hair loss [1]. The hair loss manifests as patches localized to the scalp or affecting eyebrows and eyelashes (alopecia totalis [AT]) or all hair-bearing areas of the body (alopecia universalis [AU]) [2]. AA affects approximately 2% of the general population at some point in their lifetime [1]. It is associated with multiple comorbidities, including atopic dermatitis (AD), hypothyroidism, psoriasis, vitamin D deficiency, and anemia [3,4]. Additionally, it can be psychologically burdensome to the patient as it is known to cause significant emotional stress and low self-esteem [5,6]. In some cases, this burden results in clinical manifestations of anxiety or depression [2].

A large factor in the development of emotional and psychological distress in patients with AA is societal stigmatization [7]. Stigmatization is the state of being isolated, marginalized, and ignored by the general population because of a disease or the presence of a degrading sign [7]. Patients with AA are subjected to stigmatization as their physical appearance is significantly altered by the hair loss [7]. Patients with more extensive forms of AA, such as AT and AU, may experience a greater burden [5]. This noticeable change in physical appearance causes people to view them differently, and potentially treat them differently. As a means of preventing stigmatization, patients with AA may try to conceal their hair loss. However, those with extensive hair loss may not be able to conceal it to their liking, which can cause incredible anxiety when they are faced with the task of being in public spaces or around other people. The full impact of the disease can be underestimated in clinical practice [8]. This may be due to the patients’ hesitancy to discuss their feelings with their clinicians or the clinicians’ inability to properly address the issue. This exacerbates the burden of disease as patients may feel as though their feelings are not validated by their health care providers. The emotional distress caused by stigmatization and other factors contribute to the overall burden of AA on the patients’ quality of life (QoL). Studies on the impact of AA on patients’ QoL have demonstrated that the burden often continues into adulthood [9,10]. The concept of QoL is quite subjective and multifaceted, and thus, many definitions exist [11]. For the purposes of this study, the QoL is defined as physical, emotional, and psychological well-being.

### Rationale and Objectives

There is a lack of Canadian data on the impact of AA on patients’ QoL in both adult and pediatric populations as well as the caregivers of pediatric patients. We developed a Canada-wide online survey to gather more data from patients and caregivers to help describe the disease burden. The results of this study will equip clinicians with the knowledge to actively address the burden of AA on patients and their caregivers, with the hope of improving their QoL.

## Methods

### Online Survey Development

We created 4 surveys for the following sample groups: patients 5-11 years of age (40 questions), patients 12-17 years of age (43 questions), patients 18 years of age and older (74 questions), and caregivers of children (<18 years of age) with AA (18 questions). Eligibility criteria were defined as individuals living in Canada aged 5 years or older who were clinically diagnosed with AA or the caregivers of a child clinically diagnosed with AA living in Canada. Caregivers were defined as any parent (biological or other) of a child (<18 years of age) with AA. The surveys contained questions created by the authors, as well as established clinical questionnaires. The format of the questionnaires included multiple-choice questions, yes/no questions, Likert scales, and open-ended questions. Questions created by the authors aimed to collect information about participant demographics (age and sex), history of AA (age at diagnosis, subtype, and treatments used), and the psychosocial and economic burden of the disease. The clinical questionnaires included were the Children’s Dermatology Life Quality Index (CDLQI), the Dermatology Life Quality Index (DLQI), the Hospital Anxiety and Depression Scale (HADS), and the Adjustment Disorder New Module-20 (ADNM-20).

### Validated Assessment Tools

The CDLQI questionnaire has 10 questions and is used to measure the impact of any skin disease on the lives of children aged 4-16 years [12]. The scoring of each question ranges from 3 (very much) to 0 (no impact) [12]. The total score falls into 5 categories: no effect on the child’s life (0-1), small effect on the child’s life (2-6), moderate effect on the child’s life (7-12), very large effect (13-18), and extremely large effect (19-30) [13]. In adult patients (18 years and older), the DLQI questionnaire is used [14]. The DLQI categorizes their final scores in the same way as the CDLQI but has slightly different cut-off values [15]. The HADS questionnaire is a self-assessment scale used for detecting states of depression and anxiety in a hospital medical outpatient clinic setting [16]. The HADS comprises two 7-question subscales, one targeting anxiety (HADS-A) and the other targeting depression (HADS-D) [16]. Scores for each question range from 0 (no effect) to 3 (large effect) [16]. The total score falls into 3 categories: normal (0-7), borderline abnormal (8-10), and abnormal (11-12) [16]. The ADNM-20 questionnaire has 20 questions and is used to assess the risk of an adjustment disorder diagnosis in adults [17]. The scoring of each question ranges from 1 (never) to 4 (often) [17]. The total score indicates the respondents’ risk of an adjustment disorder diagnosis, with a score of 48 or greater indicating high risk [17].

### Ethical Considerations

Ethical approval was granted through the University of Toronto (REB #00040364). Participants were required to provide written informed consent prior to completion of the surveys.

### Online Survey Dissemination

The surveys were uploaded to the SurveyMonkey platform, and the associated links were disseminated through the Canadian Alopecia Areata Foundation (CANAAF) website and to dermatologists across Canada. The surveys were completed anonymously by respondents over a period of 2 months (April-May 2021). Respondents younger than 12 years of age were required to complete the survey under the supervision of their caregivers. Respondents did not receive a monetary reward for completing the surveys.

### Data Analysis

After 2 months of data collection, the study was closed and the collected data were analyzed. Numerical data were analyzed quantitatively using Microsoft Excel version 2109. Qualitative data, namely free-text responses, were analyzed descriptively, and the most common responses were reported.

## Results

### Demographic Results

A total of 129 patients and 15 caregivers completed the surveys. The survey completion rates were 91.3% (n=105) for adult respondents, 92.9% (n=13) for pediatric respondents, and 80% (n=12) for caregiver respondents. In total, 115 (89.1%) of these 129 patients were 18 years of age and older, and 14 (10.9%) of these patients were pediatric (less than 18 years old). The mean age of pediatric patients was 13.2 (SD 3.6) years, with 13 (92.9%) being female and 1 (7.1%) being male (Table 1). The mean age of adult patients was 44.2 (SD 15.6) years, with 100 (87%) being female and 5 (13%) being male (Table 1). The mean age at diagnosis was 27.6 (SD 19.0) years for adult patients and 7.5 (SD 4.7) years for pediatric patients. The mean disease duration was 16.5 (SD 13.8) years for adult patients and 5.7 (SD 4.4) years for pediatric patients. AA affecting the scalp only and AU were the most prevalent subtypes of AA in both pediatric and adult patients (Table 1).

**Figure 1:**
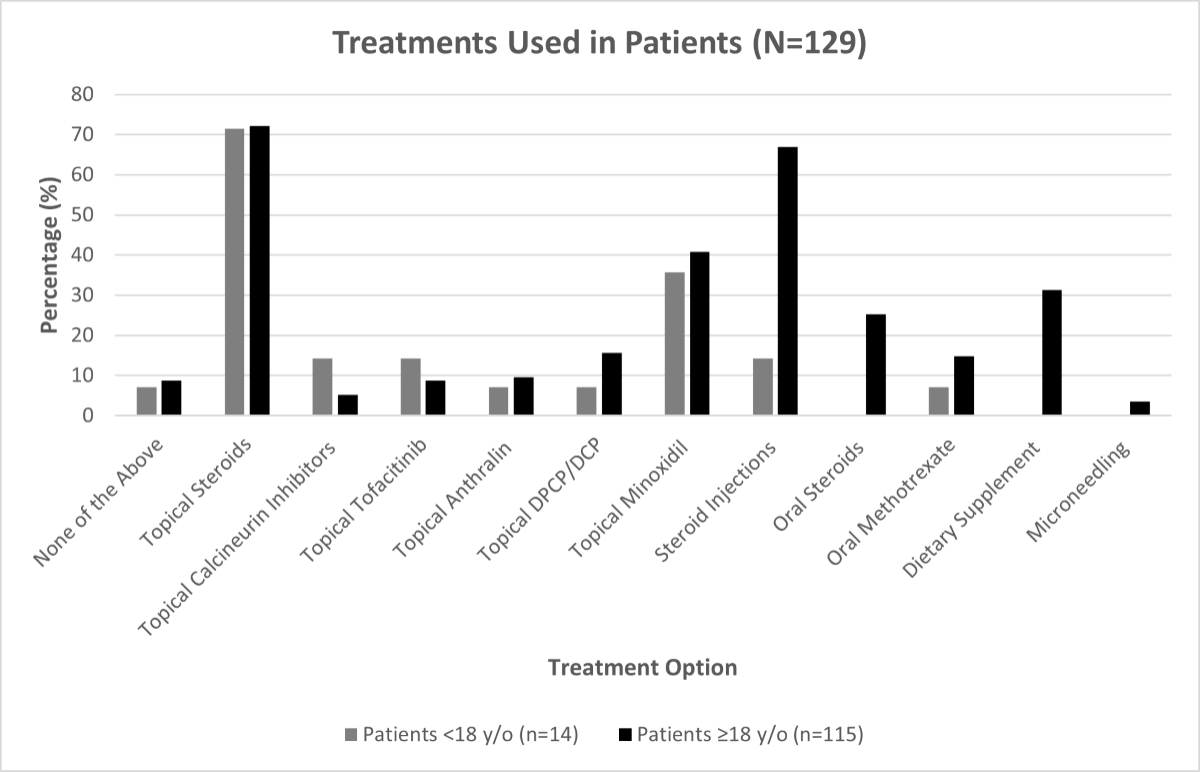
Treatments reported by respondents. The respondents were members of CANAAF or referred to the survey by a dermatologist. CANAAF: Canadian Alopecia Areata Foundation; DCP: diphencyprone; DPCP: diphenylcyclopropenone; y/o: years old.

Topical corticosteroids and intralesional corticosteroid injections were the most common treatments used in adult patients (Figure 1). In pediatric patients, topical corticosteroids and topical minoxidil were the most common treatments used (Figure 1). Vitamin D, biotin, and probiotics were the most used over-the-counter supplements by patients.

**Table 1:** Demographic characteristics of study participants.

Characteristics		Participants <18 years of age (n=14)	Participants ≥18 years of age (n=115)
**Sex, n (%)**			
	Male	1 (7.1)	15 (13.0)
	Female	13 (92.9)	100 (87.0)
**Age (years)**			
	Mean, (SD)	13.1 (3.6)	44.2 (15.6)
	Median (range)	14.5 (6-17)	43 (18-94)
**Age grouping (years), n (%)**			
	5-11	4 (28.6)	N/A^a^
	12-17	10 (71.4)	N/A
	18-30	N/A	25 (22)
	31-40	N/A	25 (22)
	41-50	N/A	20 (17)
	51-60	N/A	25 (22)
	61+	N/A	20 (17)
**AA subtype, n (%)**			
	AA^b^ (scalp only)	7 (50)	43 (37.4)
	AT^c^	2 (14.3)	17 (14.8)
	AU^d^	5 (35.7)	60 (52.2)

^a^N/A: not applicable.

^b^AA: alopecia areata. Five respondents selected AA (scalp only) plus AT or AU. Respondents were members of the Canadian Alopecia Areata Foundation (CANAAF) or referred to the survey by a dermatologist.

^c^AT: alopecia totalis, defined by loss of hair on scalp as well as eyebrows and eyelashes.

^d^AU: alopecia universalis, defined by loss of hair on areas of the body other than the head.

### Psychosocial Impact of Alopecia Areata

There was a clear impact of AA on patients’ and caregivers’ daily lives. Of the 129 patients, 123 (95%) felt uncomfortable or self-conscious about their appearance. Camouflaging hair loss with hats, scarves, and hairpieces was a common practice for 11 (78.6%) pediatric patients and 84 (73%) adult patients. Avoidance of social situations was the second highest impact of AA on daily life and was seen in 8 (57.1%) pediatric and 75 (65.2%) adult patients. Of the 15 caregivers, 6 (40%) reported this behavior in their children. Constant worry about losing the achieved hair growth was a concern for 8 (57.1%) pediatric and 75 (65.2%) adult patients. Of the 15 caregivers, 9 (60%) reported the same alopecia-related anxiety in their children. On a scale of 1-5, the mean score of caregivers’ own feelings of sadness or depression about their child’s AA was 4.0 (SD 0.9) and of their feelings of guilt or helplessness was 4.2 (SD 1.2). Caregivers’ mean satisfaction rating with the currently available AA treatment options was 1.8 (SD 0.9).

### Validated Assessment Tool Scores

**Figure 2:**
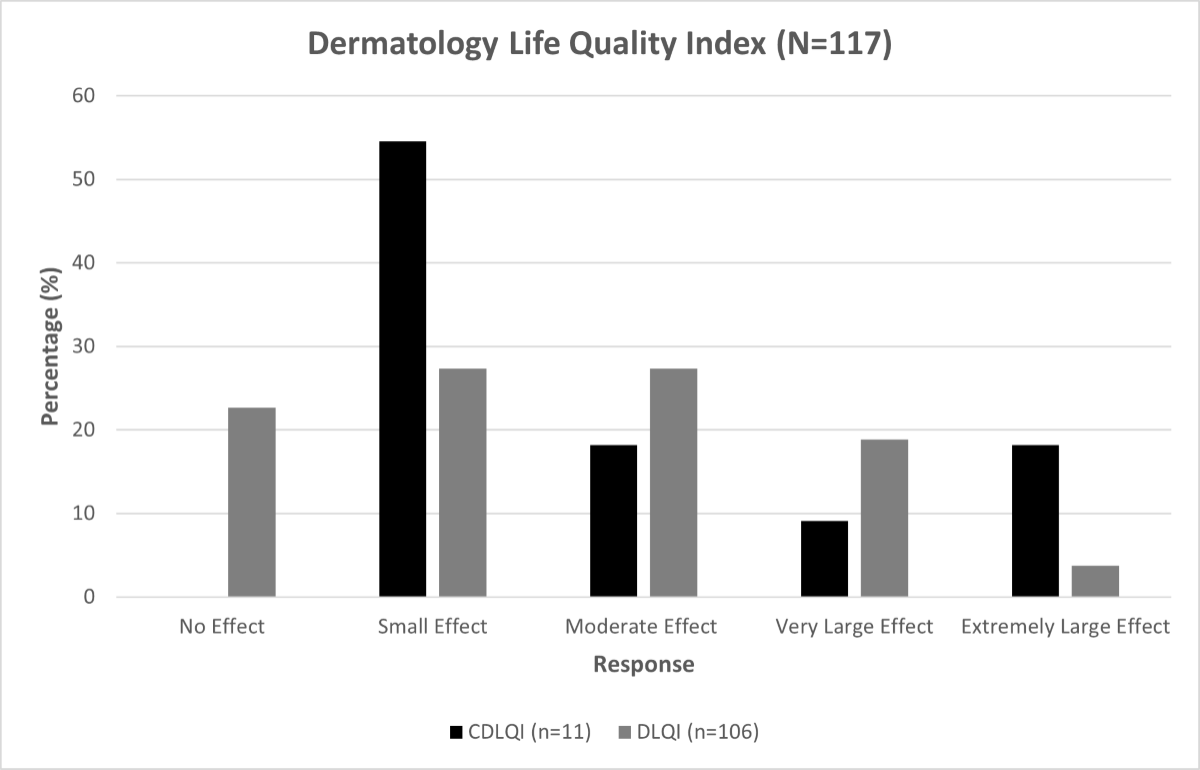
DLQI scores for all respondents. The respondents were members of CANAAF or referred to the survey by a dermatologist. The total score for both questionnaires out of 30 was categorized for impact of the dermatosis on the QoL as follows: 0-1 (no effect), 2-6 (small effect), 7-12 (moderate effect), 13-18 (very large effect), and 19-30 (extremely large effect). CANAAF: Canadian Alopecia Areata Foundation; CDLQI: Children’s Dermatology Life Quality Index; DLQI: Dermatology Life Quality Index; QoL: quality of life.

In the pediatric population, 11 (78.6%) respondents completed the CDLQI and had a mean score of 9.7 (SD 6.8), which fell within the moderate-effect range (Figure 2). In their adult counterparts, 106 (92.2%) respondents completed the DLQI section of the survey and had a mean score of 6.7 (SD 5.7), which also fell within the moderate-effect range (Figure 2).

**Figure 3:**
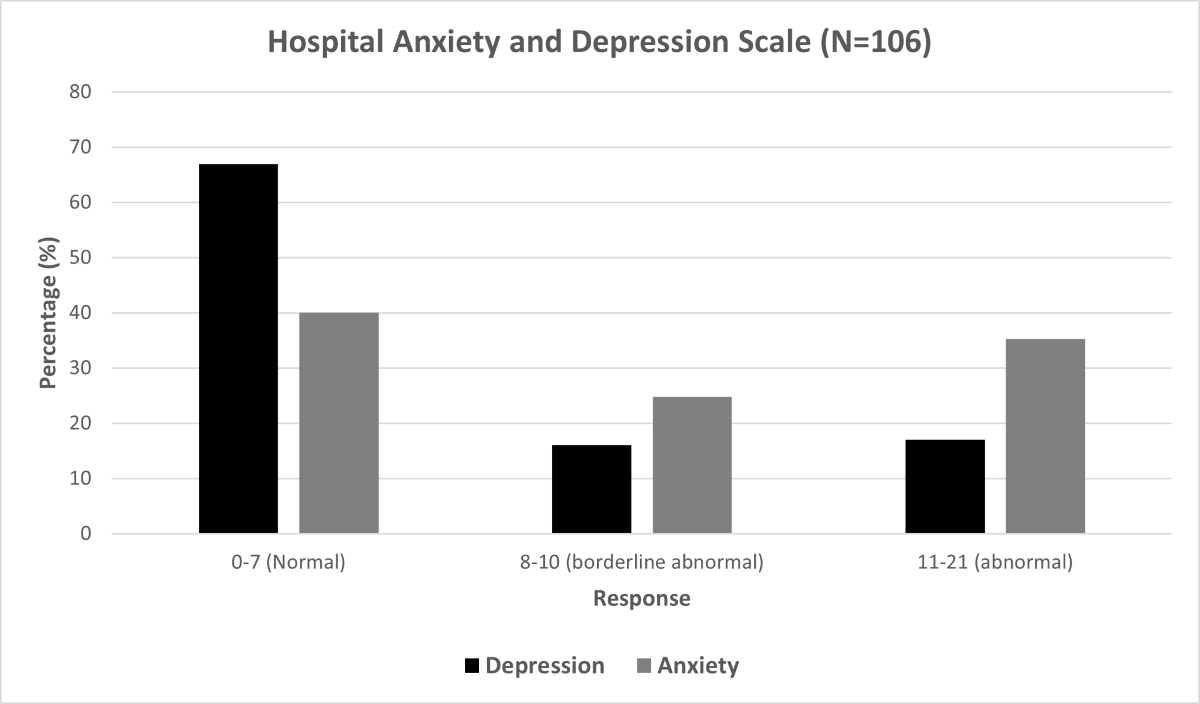
HADS scores for all respondents. The respondents were members of CANAAF or referred to the survey by a dermatologist. Both HADS-A and HADS-D scales had a total possible score of 21 from 7 questions. Results were categorized based on the total score as follows: normal (0-7), borderline abnormal (8-10), and abnormal (11-21). CANAAF: Canadian Alopecia Areata Foundation; HADS: Hospital Anxiety and Depression Scale; HADS-A: Hospital Anxiety and Depression Scale-Anxiety; HADS-D: Hospital Anxiety and Depression Scale-Depression.

Of the 115 adult respondents, 106 (92.2%) completed the ADNM-20 portion of the survey. The mean score for the ADNM-20 was 49.4 (SD 13.7), which indicated a high risk of an adjustment disorder diagnosis. In addition, 106 (92.2%) adult respondents completed the HADS-A and HADS-D portions of the survey. The mean HADS scores were 9.0 (SD 5.0) for anxiety and 5.5 (SD 4.3) for depression. The mean HADS-A score fell within the borderline abnormal range, compared to the mean HADS-D score, which fell within the normal range (Figure 3).

### Financial Impact of Alopecia Areata

Adolescent (12-17 years of age) and adult patients were asked to describe how much money they spent on eyebrow microblading and their satisfaction with the results (n=67, 51.9%). The cost of an initial microblading session ranged from CA $200-$2500 (US $146-$1825) and CA $150-$500 (US $109.5-$365) for subsequent sessions. Of the 67 patients, 54 (80.6%) were satisfied with their results. Patients described needing at least 1 or 2 microblading sessions per year to maintain cosmetically favorable results. The cost of microblading sessions and the need for continuous touch-ups were a source of frustration and financial burden.

Adult and adolescent patients were then asked to describe how much money they spent on hairpieces and whether they found them helpful (n=103, 79.8%). The cost of hairpieces ranged from CA $150-$7000 (US $109.5-$5110). Of the 103 patients, 71 (68.9%) found their hairpieces helpful, while 11 (10.7%) patients reported the hairpieces to be uncomfortable on their scalp and thus not a helpful way to conceal their hair loss. Patients reported that their hairpieces lasted 1-3 years before needing to be replaced.

### Use of Support Groups and Open-Ended Responses

Adult patients described support groups as safe spaces to express themselves and seek comfort while coping with their AA. On a Likert scale of 1-5, the mean rating of the helpfulness of both in-person and online support groups was 3.7 (SD 1.2) for adult patients. On the same scale, the mean rating for pediatric patients was 4.0 (SD 1.5) for in-person support groups and 2.8 (SD 1.5) for online support groups. Pediatric patients described support groups as fun, and they enjoyed participating in interactive activities with other youth diagnosed with AA. Both adult and pediatric patients appreciated being able to talk openly about their AA with other patients.

Patients and caregivers were asked to share any additional input they felt was valuable for us to know, and 93 (72.1%) of 129 patients and 11 (72.1%) of 15 caregivers provided free-text responses to this open-ended question. A common theme was the desire for their clinicians to provide them with more information about AA at the time of diagnosis, specifically regarding prognosis and alternative treatment options. Patients felt they were left to seek this information on their own, which was both time-consuming and emotionally taxing for them. The desire to connect sooner with support groups, such as CANAAF, was also echoed by many patients and caregivers who had wished their physicians provided them with these resources.

## Discussion

### Principal Findings

This online study confirms the burden of AA on Canadian patients’ and caregivers’ QoL. To date, this is the first Canada-wide online study of its kind. Our results demonstrated a negative financial, emotional, and psychosocial burden of AA on respondents’ daily lives. From a financial perspective, respondents reported spending several hundred to thousands of dollars yearly on cosmetic cover-ups, such as hairpieces and eyebrow microblading. From an emotional and psychosocial standpoint, respondents reported pervasive feelings of anxiety and depression that affected their ability to function as they did prior to their diagnosis of AA. Difficulty coping with AA was common among respondents, and the results of the ADNM-20 questionnaire reported a high risk of an adjustment disorder diagnosis in approximately 62% of adult patients. This is the first study to use the ADNM-20 questionnaire to assess for the risk of adjustment disorder in adult patients with AA. Adjustment disorder is a psychological reaction to a traumatic psychosocial stressor, resulting in the development of clinically significant emotional distress [18]. It has been attributed as an aggravating factor for the development of self-inflicted hair loss disorders, such as trichotillomania [19], and was reported to be the most common psychiatric comorbidity of AA by Ruiz-Doblado et al [20]. Our work has set the foundation for further Canadian studies on the association of adjustment disorder and AA, as well as other appearance-altering dermatological disorders, such as vitiligo. The online nature of the study allowed us to reach a more diverse group of patients who otherwise would have been missed using alternative formats, such as a clinic-based study. Most importantly, the results of our study have the potential to influence evidence-based care both in Canada and worldwide. For example, many patients and caregivers reported the need for more education on AA during their medical appointments, specifically as it relates to alternative treatment options and prognosis. With this information, providers may choose to allocate more time to patient education during their consultations. The emotional and psychosocial burden of disease reported by patients may also signal the need to include referral to mental health care services in the clinical management of AA.

### Comparison With Prior Studies on Alopecia Areata

The burden of AA on patients’ QoL has been previously described in studies outside of Canada [10,21,22]. A study conducted by Shi et al [23] revealed that close to 50% of patients with AA experience poor health-related QoL. Patients with more advanced forms of AA, such AT and AU, tend to have a worse QoL [24]. The mean DLQI score of our respondents was 6.7, which reflects a moderate effect of AA on patients’ QoL. This was slightly higher than the mean score of 6.3 reported by Rencz et al [25] and lower than the mean score of 7.7 reported by Liu et al [26], both of which also fall within the moderate-effect range. In our pediatric respondents, the mean CDLQI score was 9.7, which also reflects a moderate effect of AA on the QoL. This is higher than the mean score of 4.4 reported by Putterman et al [27] and drastically higher than the mean score of 2.25 reported by Vélez-Muñiz et al [28]. The large variation in mean CDLQI scores is likely due to our small pediatric population compared to the population sizes in the referenced studies. The prevalence of anxiety and depression in adults with AA can be assessed using the HADS questionnaire. The mean HADS-A score of our respondents was 9.0, which is considered borderline abnormal, and the mean HADS-D score was 5.5, which is considered normal. These values were similar to the mean scores reported by Titeca et al [29], which were 7.9 for HADS-A and 5.4 for HADS-D. A pattern can be observed in the HADS scores of our respondents and the scores found in the literature, where the HADS-A score is typically borderline abnormal or abnormal, whereas the HADS-D scores are generally normal. The high HADS-A scores can be explained by the high levels of anxiety that patients with AA experience, particularly early in their diagnosis. Much of the anxiety is social, and patients fear unpleasant social encounters with people, such as being stared at, asked intrusive questions, or being harassed. Despite the lower HADS-D scores, it is known that patients with AA experience depression at higher rates than the general public [30], which may be explained by the feelings of hopelessness and social isolation. The ADNM-20 has not been used in any other AA QoL studies to date, so no comparisons to other AA QoL studies could be made.

### Comparison With Prior Studies on Other Dermatoses

Compared to other dermatological diseases, AA appears to be less burdensome to the patient, likely due to the absence of physical symptoms, such as itch or pain. The disease most similar to AA with respect to pathogenesis and psychosocial burden is vitiligo, which manifests with disfiguring loss of skin pigmentation. A study of 100 vitiligo patients by Mishra et al [31] reported a mean DLQI score of 6.86, which is minutely higher than our mean score of 6.7. With respect to anxiety and depression, a study of vitiligo patients conducted by Ajose et al [32] reported mean HADS scores of 7.73 for anxiety and 6.18 for depression. As with the HADS scores for patients with AA, we see that the HADS scores for anxiety are higher than those of depression in vitiligo patients. Unlike AA and vitiligo, AD, an inflammatory skin disease, is notably much more burdensome for patients. Patients with AD experience chronic itching and inflammation of the skin and report much higher DLQI/CDLQI scores as a result. A systematic review by Basra et al [33] found that AD patients reported a mean DLQI score of 11.2, which is significantly higher than our mean score of 6.3. The same trend was seen in the pediatric AD population in a study conducted by Weidinger et al [34], which reported a mean CDLQI score of 14.5. The ADNM-20 questionnaire has not been used to assess for the risk of an adjustment disorder diagnosis in other dermatological disease QoL studies, and thus a comparison with our results could not be made.

### Limitations

A limitation of this study was that we relied on anonymous respondents’ self-reported AA and were not able to formally confirm their diagnoses. Most members of CANAAF are referred to the organization by their physicians, and only dermatologists were provided with information about the survey to give their patients. Thus, we felt that respondents were unlikely to have another diagnosis. Moreover, most members of CANAAF are female, which is reflected in our low number of male respondents. This is a potential source of bias and may impact the generalizability of our results. Another limitation of this study is that the surveys did not contain an AA-specific instrument, such as the Alopecia Areata Symptom Impact Scale (AASIS). However, the CDLQI/DLQI, HADS, and ADNM-20 are all validated and used routinely in clinical practice. Respondents 17 years of age completed the CDLQI despite it not being validated for their age. However, a study by van Geel et al. [35] found that DLQI and CDLQI scores were closely related in 16- and 17-year-olds; thus, we believe the resultant data are still much valuable. Due to the long duration of the surveys, respondent fatigue and resultant bias must also be considered, given the moderate length of the surveys. To mitigate this, surveys were not timed, and respondents got an opportunity to take breaks, if desired. We also had lower numbers of young children and caregivers respond, which may be due to their apprehension to answer sensitive questions in an online format. Finally, we did not analyze the impact of disease severity or patient characteristics on the disease burden because of the sample size and lack of detailed disease data.

### Conclusion

Despite limitations, the results of this first-of-its-kind Canadian survey have set the stage for further investigations on the epidemiology of AA and its impact on patients’ QoL in Canada.

## Abbreviations

AA: alopecia areata
AD: atopic dermatitis
ADNM-20: Adjustment Disorder New Module-20
AT: alopecia totalis
AU: alopecia universalis
CANAAF: Canadian Alopecia Areata Foundation
CDLQI: Children’s Dermatology Life Quality Index
DLQI: Dermatology Life Quality Index
HADS: Hospital Anxiety and Depression Scale
HADS-A: Hospital Anxiety and Depression Scale-Anxiety
HADS-D: Hospital Anxiety and Depression Scale-Depression
QoL: quality of life
